# Mortality Trends from Acute MI with Underlying CKD in the US from 1999 to 2020: A Cross-Sectional Analysis of the CDC WONDER Database

**DOI:** 10.1007/s11255-025-04720-x

**Published:** 2025-08-25

**Authors:** Rayyan Nabi, Muhammad Hussain Mansoor, Muhammad Hassan Shahbaz, Maryam Kharal, Noor Fatima, Syeda Tayyaba Munir, Sana Shahbaz, Bariqa Mujeeb, Rauha Fatima, Kirsh, Muhammad Salman Nadeem, Syeda Tahira Munir, Abdullah Nawaz Rai, Raheel Ahmed

**Affiliations:** 1 Islamic International Medical College, Riphah International University, Islamabad, Pakistan; 2 Nishtar Medical University, Multan, Pakistan; 3 National Health Services, London, UK; 4 Ziauddin Medical College, Karachi, Pakistan; 5 Services Hospital, Lahore, Pakistan; 6 National Heart and Lung Institute, Imperial College London, London, UK

**Keywords:** Chronic kidney disease, Acute myocardial infarction, Mortality trends, Health disparities, CDC WONDER

## Abstract

**Purpose:**

Chronic kidney disease increases the risk of acute myocardial infarction and worsens outcomes. Despite advances in care, mortality rate disparities regarding gender, race and socioeconomic status persist. This study examines trends in acute myocardial infarction-related mortality among chronic kidney disease patients in the United States from 1999 to 2020.

**Methods:**

We analyzed mortality data from the CDC WONDER database, identifying deaths with acute myocardial infarction (International Classification of Diseases, 10th Revision [ICD-10] codes I21–I22) as the underlying cause and chronic kidney disease (ICD-10 N18) as a contributing cause. Age-adjusted mortality rates per 100,000, standardized to the 2000 US population, were calculated and stratified by sex, race/ethnicity, urbanization, and state. Trends were assessed using Joinpoint regression to estimate annual percentage change with 95% confidence intervals.

**Results:**

A total of 72,780 acute myocardial infarction-related deaths in chronic kidney disease patients aged 25–85 years occurred from 1999 to 2020. The age adjusted mortality rate declined from 2.46 in 1999 to 0.99 in 2020 (annual percent change: –4.09; 95% CI –5.54 to –2.60). Men had higher mortality than women (age adjusted mortality rate: 2.11 vs. 1.13). Non-Hispanic Black individuals had the highest age adjusted mortality rate (2.90). Nonmetropolitan areas showed higher rates than metropolitan areas (1.63 vs. 1.51). A significant increase in mortality was observed among Hispanic patients from 2015 to 2020 (annual percent change: 8.15; 95% CI 1.13–15.65).

**Conclusions:**

Acute myocardial infarction-related mortality in chronic kidney disease patients has declined overall, but persistent disparities by sex, race/ethnicity, and geography highlight the need for continued efforts and targeted interventions in this high-risk population.

**Supplementary Information:**

The online version contains supplementary material available at 10.1007/s11255-025-04720-x.

## Introduction

The worldwide rise in the elderly population has caused a parallel surge in individuals with chronic kidney disease (CKD) requiring emergency hospital care for disease-related complications. CKD, by definition, is an abnormality in kidney function or structure (or both) present for more than 3 months with associated health implications. The estimated global prevalence of CKD is 13.4% (11.7–15.1%) [1]. It imposes a considerable economic burden, particularly related to end-stage kidney disease (ESKD) management, like renal replacement therapy and transplantation.

Research also indicates that CKD significantly elevates the risk of coronary artery disease (CAD). Acute myocardial infarction which comes under the category of coronary artery disease is one of the leading causes of death worldwide. In 2019, an estimated 17.9 million people (about the population of New York) died from cardiovascular diseases (CVDs), representing one-third of all global deaths [2].

According to scientific studies, chronic kidney disease (CKD) produces a persistent systemic inflammatory setting that significantly affects the structure and function of the heart and arteries. The formation of atherosclerotic plaques, vascular calcification, accelerated vascular aging, myocardial fibrosis, and cardiac valve calcification are all significantly influenced by persistent inflammation. Because of these pathological changes, the cardiovascular system ages more quickly [3], making CKD patients particularly vulnerable to cardiovascular issues. Hence, it is very common for patients with cardiovascular presentations to come to A&E with CKD as one of their comorbidities.

About 35–40% of deaths in CKD patients are attributable to CVD, which continues to be the leading cause of death in this population [4]. On the other hand, the mortality rate is higher for people with CAD who also have CKD than those without CKD, suggesting that having both illnesses affects patient outcomes.

Studies have shown that patients who suffer from myocardial infarction and have underlying CKD often receive less intensive treatment compared to those without CKD. This difference in treatment approaches is a contributing factor to the higher mortality rates observed in CKD patients. Particularly, patients with a low estimated glomerular filtration rate (eGFR) are less likely to undergo invasive procedures than those with preserved eGFR. Although treatment methods are improving with time, disparities persist, although to a lesser extent in patients with ST-elevation myocardial infarction (STEMI) who undergo primary percutaneous coronary intervention. However, reduced eGFR remains associated with poorer outcomes following acute myocardial infarction (AMI) [5], emphasizing the ongoing difficulties in managing these high-risk patients.

The aim of this study is to investigate mortality trends in CKD patients who experience acute myocardial infarction by analyzing data that includes various factors, including sex, ethnicity, geographic location, and degree of urbanization. By looking at these influencing elements, we aim to gain a deeper understanding of these disparities and mortality outcomes within this vulnerable patient demographic.

## Methodology

### Study setting and population

Analysis for mortality trends associated with AMI in patients with CKD in the United States from 1999 to 2020 was conducted in this study. Data on this mortality trend was extorted from CDC (Centers for Disease Control and Prevention's) WONDER (Wide-Ranging Online Data for Epidemiologic Research) official database, which is based on single and multiple causes of death, with up to 20 additional causes included, along with basic demographics from the death certificates [6]. The Multiple Cause-of-Death Public Use Record database was utilized for focusing on the cases with AMI as an underlying cause and CKD as an associated cause of death. According to the codes from the International Statistical Classification of Diseases and Related Health Problems-10th Revision (ICD-10), CKD was indicated as N18 code and AMI was indicated as I21-I22 code. This method has been implemented in previous CDC WONDER analyses [4, 7]. No institutional review board approval was required for this study, as this analysis was based on de-identified disease surveillance data retrieved from the CDC databases and is in accordance with STROBE (Strengthening the Reporting of Observational Studies in Epidemiology) guidelines [8].

### Data abstraction

The data abstracted in our study was organized according to various demographic factors including age, sex, race/ethnicity, and location of death. 10-year age groups were examined ranging from 25 + to 85 and older [9]. Ethnicity groups were categorized into Hispanic (Latino) and Non-Hispanic (NH) (with subcategorization into White, Black/African American, American Indian/Alaskan Native, and Asian). Locations of death included inpatient facilities, outpatient clinics, emergency rooms, sudden deaths, residences, hospice/nursing homes, long-term care facilities, and other unspecified places. Mortality trend was also stratified by geographic census regions (as per the United States Census Bureau), by states and by level of urbanization, as Urban metropolitan and Rural areas (according to the 2013 Urban–Rural Scheme for Counties). All these categorizations have been employed in previous CDC WONDER analysis as well [9, 10].

### Statistical analysis

Crude Mortality rates (CMRs) (deaths per 100,000 person-years) and Age Adjusted Mortality Rates AAMR (deaths per 100,000 persons standardized to 2000 US population) were calculated as per CDC standards for the analysis of the demographic variables related to mortality trends associated with AMI in patients with CKD. However, data for NH Alaskan natives was excluded from analysis due to unreliable statistics. The National Cancer Institute’s Joinpoint Regression Program 4.7.0.0 was used for the determination of Annual Percent Change (APC) values for AAMR in the analysis of temporal trends in AAMR over the study period. Joinpoint regression, a segmented regression technique, identifies points of trend change (join points) by fitting log-linear models and uses permutation tests to select the optimal number of join points (up to 3 allowed), ensuring model fit. [11] APC describes the rate of change of AAMR over time, providing a suitable measure to observe trends in mortality rates with a positive value showing an increase, whereas a negative value reflects a decrease in mortality rates. The Grid Search Method (0, 2), combined with a permutation test and parametric method, was used to calculate APC values with a 95% confidence interval. A two tailed t-test was used to determine the statistical significance of APC values, with p < 0.05 deemed statistically significant.

## Results

### Annual mortality trends

From 1999 to 2020, a total of 72,780 deaths occurred due to AMI in patients with CKD aged 25–85 years. The age-adjusted mortality rate (AAMR) associated with AMI in CKD patients was 2.46 in 1999 and 0.99 in 2020. The overall decline in AAMR has been constantly significant throughout these years (APC: − 4.09; 95% CI − 5.54 to − 2.60).

### Mortality trends by sex

**Fig. 1:**
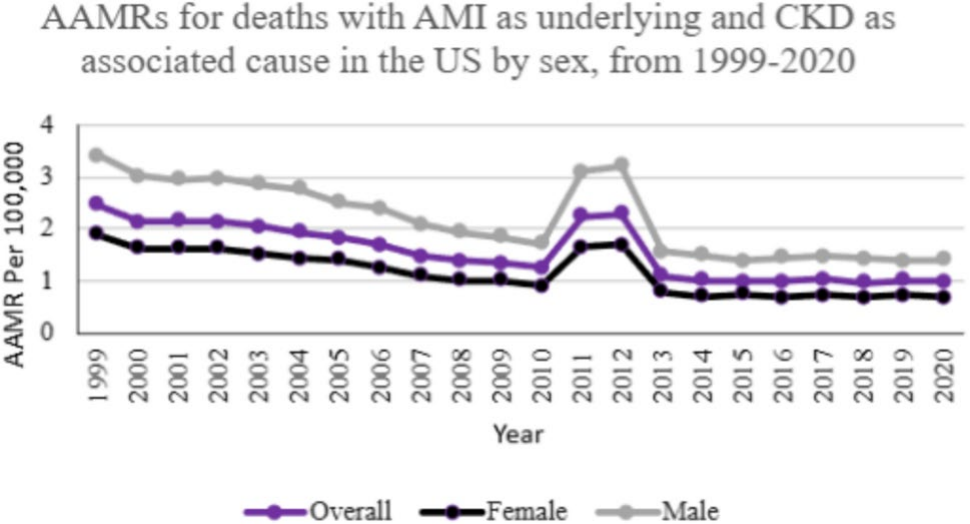
AAMRs for deaths with AMI as underlying and CKD as associated cause in the US by sex, from 1999–2020

Adult men continuously experienced higher AAMR associated with AMI in CKD patients than adult women (overall AAMR men: 2.11; 95% CI 2.09–2.13; women: 1.13; 95% CI 1.12–1.14). In 1999, AAMR was 3.41, declining to 1.42 by 2020 (APC: − 4.01; 95% CI − 5.43 to − 2.56) for men. For women, AAMR started at 1.89 in 1999 and decreased to 0.69 by 2020 (APC − 4.46; 95%CI − 5.95 to − 2.94). Both groups showed a steady yet significant decline in AAMR (Fig. 1).

### Mortality trends stratified by race/ethnicity

**Fig. 2:**
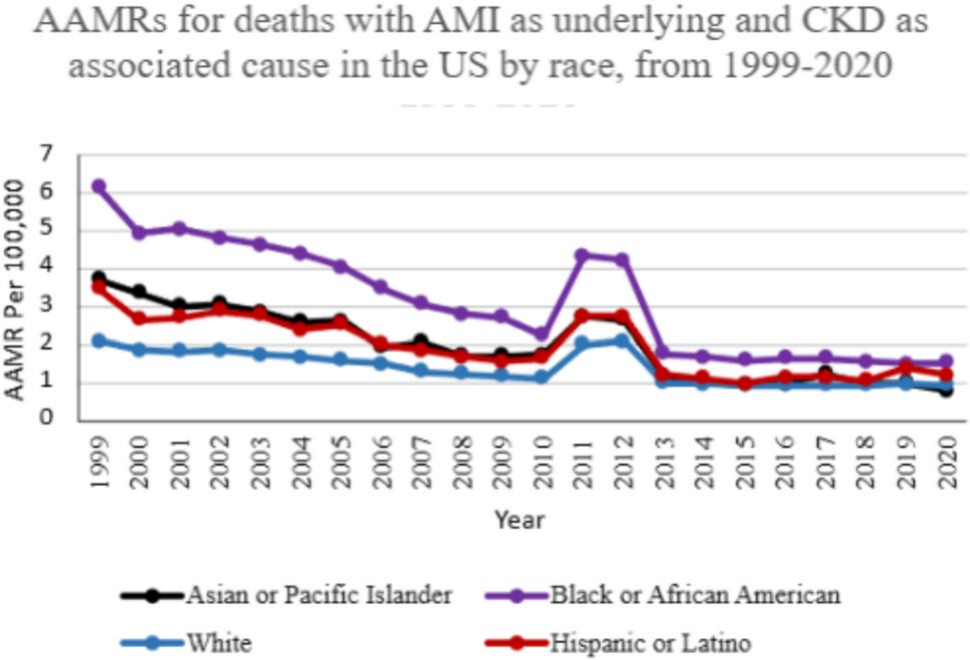
AAMRs for deaths with AMI as underlying and CKD as associated cause in the US by race, from 1999–2020

When examining AAMRs associated with AMI in CKD patients across different racial and ethnic groups, significant disparities emerge. NH Black or African American population exhibited the highest overall AAMR at 2.90, followed by Hispanic or Latino at 1.75, NH Asian or Pacific Islander at 1.69, and NH White at 1.36. Analyzing trends over time reveals that from 1999 to 2020, AAMRs generally declined for NH Black or African American (APC − 6.32; 95%CI − 7.83 to − 4.78), NH Asian or Pacific Islander (APC − 6.37; 95%CI − 7.98 to − 4.72), and NH White population (APC − 3.58; 95%CI − 5.03 to − 2.11). For the Hispanic and Latino population, age-adjusted mortality rates (AAMRs) initially showed a significant decline from 1999 to 2009 (APC − 6.74; 95% CI − 8.96 to − 4.46). This was followed by a non-significant increase from 2009 to 2012 (APC 15.88; 95% CI − 12.44 to 53.36) and a subsequent significant decline from 2012 to 2015 (APC − 29.65; 95% CI − 49.98 to − 1.04). However, from 2015 onward, AAMRs exhibited a statistically significant upward trend, continuing through the end of the study period in 2020 (APC 8.15; 95% CI 1.13 to 15.65) (Fig. 2).

## Mortality trends stratified by region

### Urbanization wise analysis

**Fig. 3:**
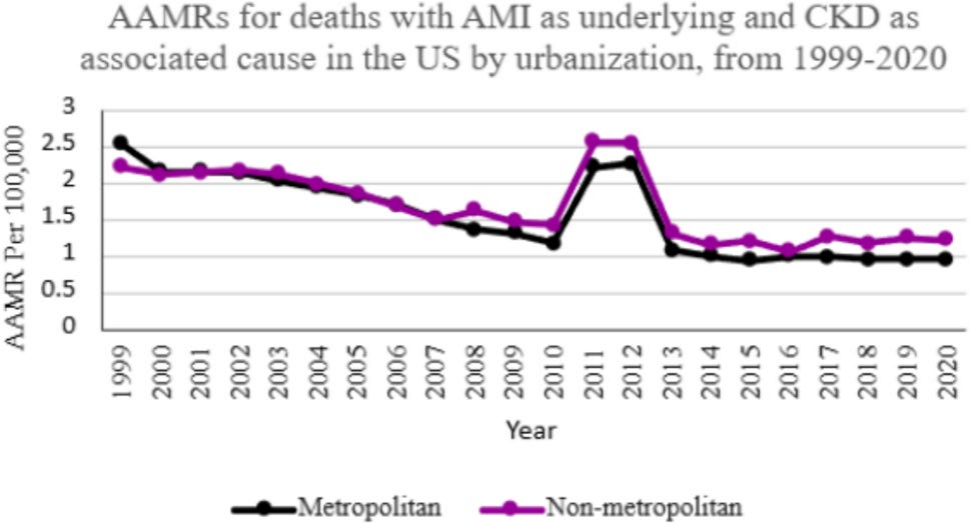
AAMRs for deaths with AMI as underlying and CKD as associated cause in the US by urbanization, from 1999–2020

Over the study period, nonmetropolitan areas showed consistently higher AAMR values from 2001 onwards, with the overall AAMR being higher (AAMR: 1.6299 [95% CI 1.6026–1.6571]) as compared to the metropolitan areas (AAMR: 1.5125 [95%CI 1.5002–1.5248]). The AAMRs of both metropolitan and nonmetropolitan areas declined from 1999 to 2020 (nonmetropolitan APC: −2.9151 [95% CI − 4.4204 to − 1.3860]; metropolitan APC − 4.3518 [95%CI − 5.8174 to − 2.8634]) (Fig. 3).

### State wise analysis

**Fig. 4:**
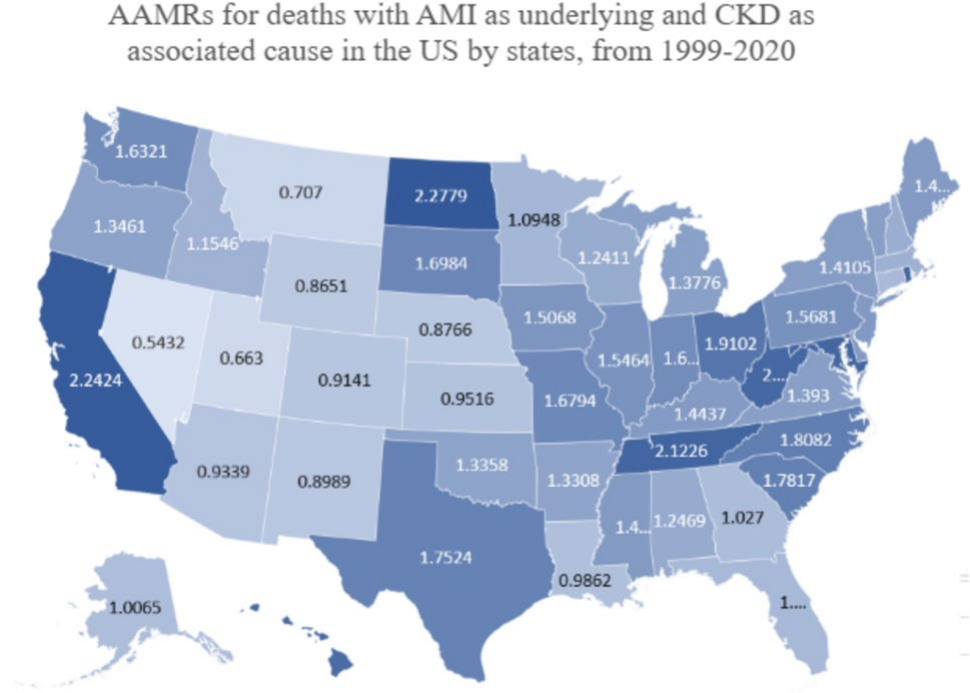
AAMRs for deaths with AMI as underlying and CKD as associated cause in the US by states, from 1999–2020

Differences exist between the AAMRs in different states with values ranging from 0.5432 in Nevada (95% CI 0.464–0.6224) to 2.3309 in the District of Columbia (95% CI 2.0033–2.6584). States in the top 90th percentile including the District of Columbia, North Dakota, California, Tennessee and Maryland exhibited AAMRs approximately three times higher compared to those in the lower 10th percentile including Nevada, Utah, Montana, Wyoming, Nebraska (Fig. 4).

## Discussion

Our study analyzed 22 years (1999–2020) of trends in AMI-associated mortality in patients with CKD in U.S. adults aged 25 and older using the CDC WONDER database. First, there was a gradual decline in the overall mortality rate from 1999 to 2020. Second, upon stratifying the data based on gender, our analysis revealed that men had higher mortality rates as compared to women. Third, we observed higher mortality rates among NH Blacks as compared to other racial groups whereas, NH Whites had the lowest AAMR. Fourth, a geographic disparity was evident, with non-metropolitan areas exhibiting higher AAMR in contrast to metropolitan areas. Fifth, our analysis demonstrated a significant state-based difference in mortality, with the District of Columbia showing the highest and Nevada showing the lowest values of AAMR.

The overall decline in mortality from 1999 to 2020 aligns with broader trends in cardiovascular disease outcomes reported in prior studies, such as Kobo et al. (2022), who observed similar reductions in CVD mortality among CKD patients using WONDER data. [4] This can be explained by improved diagnosis, proper progression, and the right referral of disease by primary care clinicians [12]. Factors contributing to the decline in AAMR range from health-promoting and prevention activities to treatment during and after an event of acute myocardial infarction in patients suffering from CKD [13]. Moreover, it is also reflected by advancements in the management strategies of AMI, including the increased use of evidence-based therapies such as statins, beta-blockers, and timely reperfusion strategies [14–17]. Studies have shown that early identification and intervention combined with public health campaigns and awareness of cardiovascular and renal health are responsible for slowing CKD progression and reducing associated renal and cardiovascular mortality [18]. These developments altogether have contributed to improved disease management, better patient outcomes, and a significant decline in AMI-related mortality in CKD patients over time. Our results also demonstrated a deviation from the overall declining trend, which was observed as a spike in AAMR values from 2011 to 2012. The sudden rise in mortality in the early 2010 s may have been driven by limited healthcare access, possibly due to barriers following the 2008 financial crisis, before improvements brought by the 2010 Affordable Care Act [19, 20].

The gender disparity observed can, in part, be attributed to the faster progression of CKD in men [21] and their increased susceptibility to cardiovascular events [22]. Men with CKD have higher systolic blood pressure compared to women, which may be a key factor in their increased risk of adverse outcomes [23]. Additionally, there are men-women differences in the detection, recognition, monitoring, referrals, and management of CKD, further contributing to the disparity seen in our study [24, 25]. Furthermore, the sex-based differences in pharmacokinetics and pharmacodynamics that are associated with changing hormonal status and aging may affect the response to different therapies for chronic kidney disease, thus being one of the probable reasons for the observed patterns in our study [26]. The protective role of estrogen in women, the detrimental effects of testosterone, along with unhealthy lifestyle habits commonly seen in men, may lead to a rapid decline in kidney function in men compared to women [27]. The greater mortality seen in men, as demonstrated by our analysis, may also be due to their greater exposure to risk factors, which include obesity, smoking, diabetes, and hypertension [28, 29]. Despite the presence of increasing literature for sex-specific recommendations in the field of nephrology, the existing knowledge gaps emphasize the value of a person-centered approach in kidney care [30].

We observed significant disparities in mortality trends among different racial and ethnic groups in the US, with African Americans aged 25 and older demonstrating the highest AMI-associated mortality rates in patients with CKD, while NH White Americans, on the other hand, showed the lowest. These results may stem from a disproportionately higher risk of heart failure and greater prevalence of comorbidities such as hypertension, diabetes, obesity, and smoking among Black patients, all of which are linked to poor cardiovascular outcomes [31–33]. This disparity can also be attributed to the higher prevalence of homelessness [34] and unemployment [35] among African Americans as compared to other racial groups. As a result, they are more likely to live in underprivileged neighborhoods [36] and are deprived of basic medical facilities, resulting in a higher mortality rate. Addressing socioeconomic inequalities, enhancing the management of comorbid conditions, and slowing disease progression are essential in reducing AMI-associated mortality rates in CKD patients among African Americans. NH Asian or Pacific Islander and Hispanic or Latino populations also demonstrated an overall decline in the mortality rates over the study period. However, our analysis identified an irregular trend in Hispanic and Latino population, likely reflecting instability among smaller subgroups. This highlights the importance of interpreting such findings with caution due to limited data availability for accurate assessment.

Our analysis also revealed regional disparity in mortality trends. In the context of urbanization, non-metropolitan areas showed the highest values of AAMR, which can be attributed to the lack of advanced medical care facilities in such areas, which contributes to the higher mortality rates [37]. Furthermore, people living in rural areas face difficulties in education, socioeconomic status, and transportation as compared to their urban counterparts [38]. While studies have shown that the introduction of telemedicine facilities has greatly improved access to medical care in rural areas [39], these advancements have been offset by the closure of hospitals in rural areas. Health policies aimed at the improvement of medical care and increasing public awareness can help bridge the gap in mortality rates between metropolitan and non-metropolitan areas. Further region-based research is required to understand such disparities and to reduce inequities in health outcomes.

State-wise analysis revealed that states in the top 90th percentile, including the District of Columbia, North Dakota, California, Tennessee, and Maryland, exhibited AAMRs approximately three times higher compared to those in the lower 10th percentile, including Nevada, Utah, Montana, Wyoming, and Nebraska. There is a relatively higher reported prevalence of hypertension, approximately 30%, among the residents of North Dakota, potentially contributing to the higher mortality rates [40]. Several studies have reported elevated rates of cardiovascular morbidity in Southern states, including the District of Columbia, Tennessee, and Maryland, as well as in Midwestern states such as North Dakota [41]. Southern states have been labeled the “stroke belt” due to their association with worse cardiovascular outcomes [42, 43]. Factors like limited healthcare access, high prevalence of risk factors, and socioeconomic challenges contribute to higher AAMR observed in California [44, 45]. Another possible underlying factor to this disparity is the uneven distribution of insurance facilities that has been observed across all states in the US. Therefore, policies promoting Medicaid expansion and more equitable coverage of all patients can help bridge the disparity gap.

Although there has been a slow drop in the mortality rates related to CKD over the span of years, it is noteworthy that even in high-income countries, it has been the ninth most predominant cause of fatality [46]. The emerging element is CKD being a reason for compromised life span in most of its affected individuals compared to the general population due to its association with cardiovascular diseases [47]. Previous studies have consistently demonstrated an increased risk of developing acute MI in CKD patients with progressively worsening outcomes with increasing severity of CKD [5, 48]. Even mild renal dysfunction has been considered to be a major risk factor for cardiovascular complications after MI [49]. Notably, AMI patients exhibit a significantly elevated prevalence (40%) of chronic kidney disease compared to the general population (11–13%), thus highlighting CKD as a common comorbidity and frequent contributing factor to acute MI. [1, 48, 50, 51] This data underscores the importance of community-level prevention, such as the primary prevention of major risk factors including hypertension, obesity, and diabetes. Regarding CKD, the holistic approach makes prominent the essentiality to reduce and prevent oxidative stress, use of nephrotoxic drugs, kidney stones, acute kidney injuries, and other environmental exposures [52]. Local epidemiology emphasizes the use of safe water, proper sanitation availability, and vector control measures to prevent infections leading to CKD. Public healthcare strategies, including screening for early detection, must be implemented. Funding and advocacy for further research and public health investments must be encouraged. Proper sets of clinical care, awareness, and education must be ensured in CKD-inflicted areas. Also, genetic studies can help evaluate the inheritable element affecting different populations towards specific environmental predisposing factors [53]

## Limitations

Our analysis provides valuable insights into the mortality trends due to AMI in CKD patients. There are several notable strengths and limitations associated with our study. First, the study’s design as a retrospective ecological study of aggregate death counts does not permit causal inferences to be made at the individual level and is subject to ecological fallacy. Second, the CDC WONDER database hides counts fewer than 10 to preserve the deidentification of data and flags rates based on fewer than 20 deaths as “unreliable,” resulting in skewed data that limits the analysis of true trends of small subgroups such as NH Alaskan natives'mortality due to AMI in CKD patients. Third, the reliance of data on clinician-entered ICD codes makes it susceptible to misclassification due to the absence of laboratory or chart validation. It is important to note that these inherent limitations of the database could potentially lead to underrepresentation of true burdens of AMI-associated mortality in patients with CKD. Fourth, CDC WONDER provides aggregate data that does not account for individual-level confounding variables such as socioeconomic status, insurance type, and clinical severity scores, which limits multivariable adjustment for confounders affecting both AMI and CKD outcomes. Fifth, although JoinPoint is unable to measure the extent of bias introduced by unmeasured confounders or data suppression, it is still a strong tool for identifying inflection points in mortality trends, enabling us to effectively observe mortality trends over time. Finally, the reliability of the data analyzed may further be compromised due to the undiagnosed or asymptomatic cases going unreported. Despite these limitations, the large-scale, longitudinal nature of the dataset provided by CDC WONDER allows us to effectively observe and analyze demographical trends at a population level.

## Conclusion

To summarize, our results demonstrate a significant overall decline in AMI-associated mortality in patients with CKD. Highest mortality rates were observed in NH Black adults, men, and individuals living in non-metropolitan areas. These disparities emphasize the critical need for public health initiatives to enhance preventive care, and healthcare access to improve AMI-associated outcomes in CKD patients. Further research, including meta-analyses and individual-level studies, is warranted to contribute to our findings and explore health inequities.

## Supplementary Information

Below is the link to the electronic supplementary material.

Supplementary file1 (DOCX 60 KB)

## Data Availability

Data is provided within the manuscript or supplementary information files.
