# Supplementary material for: Mortality Trends from Acute MI with Underlying CKD in the US from 1999 to 2020: A Cross-Sectional Analysis of the CDC WONDER Database
Source: Int Urol Nephrol. 2025 Aug 25;58(2):653–60. doi: 10.1007/s11255-025-04720-x (PMC12864192; doi:10.1007/s11255-025-04720-x)
Supplement: Supplementary file 1 — Supplementary file1 (DOCX 60 KB) [file 11255_2025_4720_MOESM1_ESM.docx]

**Supplementary File, CDC – AMI & CKD**

| **Table 1: Frequency and age adjusted mortality rates per 100,000 deaths in adults aged 25-85+ in the United States, 1999 to 2020** | | | |
| --- | --- | --- | --- |
|  | **Deaths** | **Population** | **Overall AAMR per 100,000 deaths (95% CI)** |
| Entire Cohort excluding Hispanic | 72780 | 4473854489 | 1.54 (1.52 – 1.55) |
| **Sex** |  |  |  |
| Male | 41452 | 2154556911 | 2.12 (2.09 – 2.14) |
| Female | 31328 | 2319297578 | 1.13 (1.12 – 1.15) |
| **Race** |  |  |  |
| NH Asian or Pacific Islander | 3050 | 245576124 | 1.69 (1.62 – 1.75) |
| NH Black or African American | 13182 | 546447527 | 2.90 (2.85 – 2.95) |
| NH White | 55967 | 3631977407 | 1.36 (1.35 – 1.38) |
| NH American Indian or Alaska Native | 581 | 49853431 | 1.71 (1.56 – 1.86) |
| Hispanic | 6184 | 589350210 | 1.76 (1.71 – 1.80) |
| **Urbanization** |  |  |  |
| Metropolitan | 58858 | 3795213822 | 1.51 (1.5 – 1.52) |
| Nonmetropolitan | 13922 | 678634169 | 1.63 (1.60 – 1.66) |

**Supplementary Table 1: Frequency and age adjusted mortality rates per 100,000 deaths in adults aged 25-85+ in the United States, 1999 to 2020**

| **Table 2: Annual age adjusted mortality rates per 100,000 deaths in adults aged 25-85+ in the United States, 1999 to 2020** | |
| --- | --- |
| **Year** | **Age Adjusted Mortality Rate per 100,000 deaths (95% CI)** |
| 1999 | 2.46 (2.39 - 2.53) |
| 2000 | 2.14 (2.08 - 2.21) |
| 2001 | 2.16 (2.09 - 2.23) |
| 2002 | 2.14 (2.08 - 2.21) |
| 2003 | 2.04 (1.98 - 2.11) |
| 2004 | 1.93 (1.87 - 1.99) |
| 2005 | 1.84 (1.78 - 1.9) |
| 2006 | 1.71 (1.65 - 1.76) |
| 2007 | 1.47 (1.42 - 1.53) |
| 2008 | 1.4 (1.35 - 1.45) |
| 2009 | 1.34 (1.29 - 1.39) |
| 2010 | 1.25 (1.2 - 1.29) |
| 2011 | 2.25 (2.19 - 2.32) |
| 2012 | 2.3 (2.24 - 2.36) |
| 2013 | 1.11 (1.06 - 1.15) |
| 2014 | 1.01 (0.97 - 1.05) |
| 2015 | 1 (0.96 - 1.04) |
| 2016 | 1 (0.96 - 1.04) |
| 2017 | 1.05 (1.01 - 1.09) |
| 2018 | 0.97 (0.93 - 1.01) |
| 2019 | 1.01 (0.97 - 1.05) |
| 2020 | 1 (0.96 - 1.04) |

**Supplementary Table 2: Annual age adjusted mortality rates per 100,000 deaths in adults aged 25-85+ in the United States, 1999 to 2020**

| **Table 3: Age adjusted mortality rates per 100,000 deaths stratified by sex in adults aged 25-85+ in the United States, 1999 to 2020** | | |
| --- | --- | --- |
|  | **Age Adjusted Mortality Rate per 100,000 deaths (95% CI)** | |
| **Year** | **Female** | **Male** |
| 1999 | 1.9 (1.81 - 1.98) | 3.42 (3.28 - 3.56) |
| 2000 | 1.64 (1.56 - 1.72) | 3.01 (2.88 - 3.14) |
| 2001 | 1.62 (1.55 - 1.7) | 2.95 (2.83 - 3.08) |
| 2002 | 1.62 (1.55 - 1.7) | 2.99 (2.86 - 3.11) |
| 2003 | 1.52 (1.45 - 1.59) | 2.88 (2.75 - 3) |
| 2004 | 1.44 (1.37 - 1.51) | 2.77 (2.65 - 2.89) |
| 2005 | 1.41 (1.34 - 1.48) | 2.52 (2.4 - 2.63) |
| 2006 | 1.25 (1.18 - 1.31) | 2.4 (2.29 - 2.51) |
| 2007 | 1.1 (1.04 - 1.16) | 2.09 (1.99 - 2.19) |
| 2008 | 1.02 (0.96 - 1.08) | 1.95 (1.85 - 2.04) |
| 2009 | 1.01 (0.95 - 1.07) | 1.86 (1.77 - 1.95) |
| 2010 | 0.91 (0.86 - 0.97) | 1.73 (1.64 - 1.82) |
| 2011 | 1.66 (1.59 - 1.74) | 3.12 (3 - 3.23) |
| 2012 | 1.7 (1.63 - 1.77) | 3.21 (3.09 - 3.33) |
| 2013 | 0.8 (0.75 - 0.85) | 1.56 (1.48 - 1.64) |
| 2014 | 0.71 (0.66 - 0.75) | 1.5 (1.42 - 1.57) |
| 2015 | 0.74 (0.69 - 0.79) | 1.38 (1.31 - 1.46) |
| 2016 | 0.69 (0.65 - 0.73) | 1.45 (1.37 - 1.52) |
| 2017 | 0.72 (0.68 - 0.76) | 1.47 (1.4 - 1.55) |
| 2018 | 0.69 (0.65 - 0.73) | 1.42 (1.35 - 1.5) |
| 2019 | 0.73 (0.69 - 0.78) | 1.4 (1.33 - 1.47) |
| 2020 | 0.69 (0.65 - 0.73) | 1.42 (1.35 - 1.49) |

**Supplementary Table 3: Age adjusted mortality rates per 100,000 deaths stratified by sex in adults aged 25-85+ in the United States, 1999 to 2020**

|  |  |  |  |  |
| --- | --- | --- | --- | --- |
| **Table 4: Age adjusted mortality rates per 100,000 deaths stratified by race in adults aged 25-85+ in the United States, 1999 to 2020** | | | | |
| **Year** | **Age Adjusted Mortality Rate per 100,000 deaths (95% CI)** | | | |
|  | **NH Asian or Pacific Islander** | **Black or African American** | **NH White** | **Hispanic or Latino** |
| 1999 | 3.72 (3.1 - 4.34) | 6.13 (5.74 - 6.52) | 2.08 (2.01 - 2.15) | 3.46 (3.06 - 3.86) |
| 2000 | 3.35 (2.77 - 3.93) | 4.92 (4.56 - 5.27) | 1.84 (1.78 - 1.91) | 2.64 (2.3 - 2.98) |
| 2001 | 3.02 (2.5 - 3.53) | 5.04 (4.68 - 5.39) | 1.83 (1.76 - 1.9) | 2.69 (2.36 - 3.03) |
| 2002 | 3.06 (2.55 - 3.56) | 4.81 (4.47 - 5.15) | 1.86 (1.79 - 1.92) | 2.89 (2.55 - 3.24) |
| 2003 | 2.87 (2.39 - 3.34) | 4.61 (4.28 - 4.94) | 1.74 (1.68 - 1.81) | 2.77 (2.44 - 3.1) |
| 2004 | 2.58 (2.14 - 3.03) | 4.39 (4.07 - 4.71) | 1.67 (1.61 - 1.74) | 2.39 (2.1 - 2.68) |
| 2005 | 2.61 (2.18 - 3.04) | 4.03 (3.72 - 4.33) | 1.6 (1.54 - 1.66) | 2.53 (2.24 - 2.83) |
| 2006 | 1.95 (1.59 - 2.31) | 3.47 (3.19 - 3.75) | 1.5 (1.44 - 1.56) | 2.01 (1.76 - 2.27) |
| 2007 | 2.05 (1.69 - 2.4) | 3.05 (2.79 - 3.31) | 1.29 (1.23 - 1.34) | 1.86 (1.62 - 2.11) |
| 2008 | 1.7 (1.39 - 2.02) | 2.8 (2.55 - 3.04) | 1.23 (1.18 - 1.28) | 1.67 (1.44 - 1.89) |
| 2009 | 1.7 (1.39 - 2.01) | 2.7 (2.46 - 2.94) | 1.18 (1.13 - 1.24) | 1.57 (1.36 - 1.78) |
| 2010 | 1.74 (1.43 - 2.04) | 2.23 (2.01 - 2.44) | 1.1 (1.05 - 1.15) | 1.63 (1.42 - 1.84) |
| 2011 | 2.73 (2.36 - 3.1) | 4.32 (4.03 - 4.62) | 2 (1.94 - 2.06) | 2.74 (2.47 - 3) |
| 2012 | 2.66 (2.31 - 3.01) | 4.21 (3.93 - 4.5) | 2.09 (2.02 - 2.15) | 2.74 (2.49 - 3) |
| 2013 | 1.18 (0.96 - 1.4) | 1.77 (1.59 - 1.95) | 1 (0.96 - 1.05) | 1.19 (1.03 - 1.36) |
| 2014 | 1.02 (0.82 - 1.23) | 1.67 (1.5 - 1.84) | 0.95 (0.91 - 1) | 1.11 (0.95 - 1.26) |
| 2015 | 0.94 (0.76 - 1.13) | 1.57 (1.41 - 1.74) | 0.94 (0.9 - 0.98) | 0.95 (0.82 - 1.09) |
| 2016 | 0.96 (0.78 - 1.14) | 1.64 (1.47 - 1.8) | 0.93 (0.88 - 0.97) | 1.14 (1 - 1.29) |
| 2017 | 1.24 (1.03 - 1.44) | 1.66 (1.5 - 1.82) | 0.94 (0.9 - 0.98) | 1.14 (1 - 1.28) |
| 2018 | 1 (0.82 - 1.18) | 1.54 (1.39 - 1.7) | 0.93 (0.89 - 0.97) | 1.04 (0.91 - 1.18) |
| 2019 | 0.99 (0.82 - 1.17) | 1.5 (1.35 - 1.65) | 0.96 (0.92 - 1) | 1.37 (1.22 - 1.52) |
| 2020 | 0.8 (0.65 - 0.95) | 1.52 (1.37 - 1.67) | 0.93 (0.89 - 0.97) | 1.22 (1.08 - 1.36) |

**Supplementary Table 4: Age adjusted mortality rates per 100,000 deaths stratified by race in adults aged 25-85+ in the United States, 1999 to 2020**

| **Table 5: Age adjusted mortality rates per 100,000 deaths stratified by urbanization in adults aged 25-85+ in the United States, 1999 to 2020** | | |
| --- | --- | --- |
| **Year** | **Age Adjusted Mortality Rate per 100,000 deaths (95% CI)** | |
|  | **Metropolitan** | **Nonmetropolitan** |
| 1999 | 2.53 (2.45 - 2.61) | 2.22 (2.06 - 2.38) |
| 2000 | 2.16 (2.08 - 2.23) | 2.1 (1.95 - 2.26) |
| 2001 | 2.16 (2.08 - 2.23) | 2.14 (1.99 - 2.3) |
| 2002 | 2.14 (2.07 - 2.22) | 2.17 (2.01 - 2.33) |
| 2003 | 2.04 (1.96 - 2.11) | 2.12 (1.97 - 2.27) |
| 2004 | 1.94 (1.87 - 2.01) | 1.99 (1.84 - 2.14) |
| 2005 | 1.83 (1.76 - 1.9) | 1.85 (1.71 - 2) |
| 2006 | 1.7 (1.63 - 1.76) | 1.69 (1.56 - 1.83) |
| 2007 | 1.5 (1.44 - 1.56) | 1.49 (1.36 - 1.62) |
| 2008 | 1.36 (1.3 - 1.41) | 1.62 (1.49 - 1.75) |
| 2009 | 1.31 (1.26 - 1.36) | 1.47 (1.35 - 1.59) |
| 2010 | 1.18 (1.13 - 1.23) | 1.42 (1.3 - 1.55) |
| 2011 | 2.21 (2.14 - 2.28) | 2.56 (2.4 - 2.72) |
| 2012 | 2.26 (2.19 - 2.33) | 2.55 (2.39 - 2.71) |
| 2013 | 1.08 (1.03 - 1.12) | 1.31 (1.2 - 1.43) |
| 2014 | 1 (0.95 - 1.04) | 1.15 (1.05 - 1.26) |
| 2015 | 0.94 (0.9 - 0.99) | 1.2 (1.09 - 1.31) |
| 2016 | 1 (0.95 - 1.04) | 1.06 (0.96 - 1.15) |
| 2017 | 0.99 (0.95 - 1.03) | 1.26 (1.15 - 1.37) |
| 2018 | 0.95 (0.91 - 0.99) | 1.17 (1.07 - 1.27) |
| 2019 | 0.96 (0.92 - 1) | 1.26 (1.15 - 1.36) |
| 2020 | 0.96 (0.92 - 1) | 1.21 (1.11 - 1.32) |

**Supplementary Table 5: Age adjusted mortality rates per 100,000 deaths stratified by urbanization in adults aged 25-85+ in the United States, 1999 to 2020**

| **Table 6: State wise age adjusted mortality rates per 100,000 deaths in adults aged 25-85+ in the United States, 1999 to 2020** | |
| --- | --- |
| **State** | **AAMR per 100,000 deaths (95% CI)** |
| Alabama | 1.25 (1.17 - 1.33) |
| Alaska | 1.01 (0.76 - 1.31) |
| Arizona | 0.93 (0.87 - 0.99) |
| Arkansas | 1.33 (1.23 - 1.44) |
| California | 2.24 (2.2 - 2.28) |
| Colorado | 0.91 (0.84 - 0.99) |
| Connecticut | 1.06 (0.97 - 1.14) |
| Delaware | 1.44 (1.25 - 1.64) |
| District of Columbia | 2.33 (2 - 2.66) |
| Florida | 1.07 (1.04 - 1.1) |
| Georgia | 1.03 (0.97 - 1.08) |
| Hawaii | 2.04 (1.85 - 2.23) |
| Idaho | 1.15 (1.01 - 1.3) |
| Illinois | 1.55 (1.49 - 1.6) |
| Indiana | 1.62 (1.54 - 1.7) |
| Iowa | 1.51 (1.4 - 1.61) |
| Kansas | 0.95 (0.86 - 1.04) |
| Kentucky | 1.44 (1.35 - 1.54) |
| Louisiana | 0.99 (0.91 - 1.06) |
| Maine | 1.44 (1.29 - 1.6) |
| Maryland | 2.1 (2 - 2.2) |
| Massachusetts | 1.17 (1.1 - 1.23) |
| Michigan | 1.38 (1.32 - 1.44) |
| Minnesota | 1.09 (1.02 - 1.17) |
| Mississippi | 1.45 (1.34 - 1.57) |
| Missouri | 1.68 (1.6 - 1.76) |
| Montana | 0.71 (0.58 - 0.84) |
| Nebraska | 0.88 (0.77 - 0.99) |
| Nevada | 0.54 (0.46 - 0.62) |
| New Hampshire | 1.21 (1.06 - 1.36) |
| New Jersey | 1.46 (1.4 - 1.52) |
| New Mexico | 0.9 (0.79 - 1.01) |
| New York | 1.41 (1.37 - 1.45) |
| North Carolina | 1.81 (1.74 - 1.88) |
| North Dakota | 2.28 (2.01 - 2.55) |
| Ohio | 1.91 (1.85 - 1.97) |
| Oklahoma | 1.34 (1.24 - 1.43) |
| Oregon | 1.35 (1.25 - 1.44) |
| Pennsylvania | 1.57 (1.52 - 1.62) |
| Rhode Island | 2.09 (1.89 - 2.3) |
| South Carolina | 1.78 (1.68 - 1.88) |
| South Dakota | 1.7 (1.48 - 1.91) |
| Tennessee | 2.12 (2.03 - 2.21) |
| Texas | 1.75 (1.71 - 1.8) |
| Utah | 0.66 (0.57 - 0.76) |
| Vermont | 1.39 (1.17 - 1.62) |
| Virginia | 1.39 (1.32 - 1.46) |
| Washington | 1.63 (1.55 - 1.71) |
| West Virginia | 2.09 (1.93 - 2.24) |
| Wisconsin | 1.24 (1.17 - 1.31) |
| Wyoming | 0.87 (0.67 - 1.09) |

**Supplementary Table 6: State wise age adjusted mortality rates per 100,000 deaths in adults aged 25-85+ in the United States, 1999 to 2020**
